# Interaction of Graphene Oxide Particles and Dendrimers with Human Breast Cancer Cells by Real-Time Microscopy

**DOI:** 10.3390/pharmaceutics15122655

**Published:** 2023-11-22

**Authors:** Beatriz Fumelli Monti Ribeiro, Julyane Batista Chaves, Marcelo Medina De Souza, Artur Franz Keppler, Devaney Ribeiro Do Carmo, Gláucia M. Machado-Santelli

**Affiliations:** 1 Department of Cell and Developmental Biology, Institute of Biomedical Sciences, University of São Paulo, São Paulo 05508-000, Brazil; 2 Centre of Excellence in New Target Discovery (CENTD), Butantan Institute, São Paulo 05503-900, Brazil; 3 Centre of Natural and Human Sciences (CCNH), Federal University of ABC, São Paulo 09210-170, Brazil; 4 Department of Physics and Chemistry, Paulista State University, São Paulo 01049-010, Brazil

**Keywords:** graphene, nanoparticles internalization, real-time microscopy, breast cancer cell culture

## Abstract

Graphene oxide (GOX) has become attractive due to its unique physicochemical properties. This nanomaterial can associate with other dendrimers, making them more soluble and allowing better interaction with biomacromolecules. The present study aimed to investigate, by real-time microscopy, the behavior of human breast cancer cells exposed to particles of materials based on graphene oxide. The MCF-7 cell line was exposed to GOX, GOX associated with Polypropylenimine hexadecaamine Dendrimer, Generation 3.0—DAB-AM-16 (GOXD) and GOX associated with polypropyleneimine—PAMAM (GOXP) in the presence or absence of fetal bovine serum (FBS). GOX, GOXD and GOXP were taken up by the cells in clusters and then the clusters were fragmented into smaller ones inside the cells. Real-time microscopy showed that the presence of FBS in the culture medium could allow a more efficient internalization of graphene materials. After internalizing the materials, cells can redistribute the clumps to their daughter cells. In conclusion, the present study showed that the particles can adhere to the cell surface, favoring their internalization. The presence of FBS contributed to the formation of smaller aggregates of particles, avoiding the formation of large ones, and thus transmitted a more efficient internalization of the materials through the interaction of the particles with the cell membrane.

## 1. Introduction

Graphene is a material composed of a single layer of carbon atoms characterized by a two-dimensional structure in the form of a honeycomb [1] and has gained prominence in the academic environment because it has interesting applications. Graphene oxide, one of the derivatives of graphene, has become attractive due to its unique physicochemical properties, such as its high surface area [2] and can be used for DNA and protein detection, as well as drug delivery [3,4,5].

Another interesting aspect of these nanomaterials is their association with other dendrimers, making them more soluble and allowing a better interaction with bio macromolecules [6]. Several authors have already reported that the non-covalent conjugation of anticancer molecules with graphene oxide sheets may be the path to more targeted delivery at the cellular level and a possible escape from tumor resistance [7,8]. Structural modification aimed at solving these limitations and reducing the cytotoxicity of the material is an interesting strategy for the biological applications of graphene [9]. Among the possible structural modifications in graphene particles, the association with dendrimers, such as polyamide amine (PAMAM), can provide better cell uptake since PAMAM dendrimers have been reported to be able to associate electrostatically with lipid membranes to form amphiphilic bilayers and thus promote cell uptake [10,11,12].

Upon contact with (bio) macromolecules, the surface of these nanomaterials is slightly altered, and one of the modifications observed is the adsorption of (bio) molecules, usually proteins, to coat and encompass the structure of the nanomaterial, forming the known ‘protein corona’ [13,14]. This alteration seems to make the structure more bio-acceptable to organisms and may facilitate the internalization of material by cells [15,16,17]. Also, the composition profiles of the corona molecules differ significantly from the protein composition of the biological fluid investigated [18,19,20].

Previous studies have shown that almost all mammalian cells are capable of incorporating nanoparticles to some degree due to a series of specific uptake mechanisms [21,22,23,24]. Therefore, understanding the effects of different fluid components on the internalization of nanoparticles is a relevant way for these tools to be potentiated in biomedical applications. Thus, the objective of this work was to evaluate the behavior of human breast cancer cells exposed to particles of graphene oxide-based materials by real-time microscopy.

## 2. Materials and Methods

### 2.1. Synthesis of Materials

The synthesis process and the physicochemical characterization of the materials are described in Ribeiro et al. 2020; Fernandes et al. 2018 and Do Carmo and Fernandes, 2017 [25,26,27].

All reagents used in the preparation and functionalization processes were obtained from Sigma-Aldrich without further purification. GOX was purchased from Sigma-Aldrich (St. Louis, MO, USA). GOX was functionalized with Polypropylenimine hexadecaamine Dendrimer, Generation 3.0—DAB-AM-16 (GOXD) and with polypropyleneimine—PAMAM (MW 516.68 g/mol) (GOXP). The procedure was described in the literature [26,27] with some modifications. In a 100 mL round-bottom flask, 1.0 g of GO powder, 1.3 g of dendrimer and 0.100 g of catalyst *N*,*N*′-Dicyclohexylcarbodiimide (DCC) were added to 40 mL of methanol, followed by ultrasonication for 10 min. The dispersion was then refluxed in an inert medium (N_2_) for 24 h. Once the reaction was complete, the dispersion was roto evaporated and the obtained powder was vacuum filtered and washed exhaustively with methanol to remove the unreacted dendrimer. The final product (GOXD and GOXP powder) was dried in a vacuum oven (50 °C).

The characterization of the carbonaceous materials described here is crucial to identify possible structural and surface changes responsible for the reactivity and, consequently, for the effects of nanomaterials on the environment, as well as on human health. As a result of previous studies carried out on the carbonaceous materials evaluated here, they have already been subjected to a comprehensive analysis using a combination of analytical and spectroscopic methods [26,27,28].

### 2.2. MCF-7 Cell Culture

The MCF-7 cell line, established from the pleural effusion of breast adenocarcinoma, was acquired from the BCRJ (Duque de Caxias, Brazil). Cells were cultured in DMEM/F12 (Dulbecco’s Modified Eagle Medium: Nutrient Mixture F-12) culture medium (Sigma Chemical Co., St. Louis, MO, USA) supplemented with 10% Fetal Bovine Serum (FBS), (FBS, Atena, Rio de Janeiro, Brazil). The cell cultures were maintained at 37 °C with high relative humidity in a controlled atmosphere containing 5% CO_2_.

### 2.3. Generation of MCF-7 Cell Spheroids

The three-dimensional (3D) MCF-7 cell culture was prepared by the liquid overlay technique (LOT) in 96-well sterile cell culture plates (Kasvi, São José Dos Pinhais, Brazil). Initially, the wells of the culture plates were coated with 60 µL of 1% agarose (Sigma, São Paulo, Brazil), allowing this reagent to set at least 30 min with the lids left off the plates. In each well, 5 × 10^3^ cells were seeded, and 200 µL of DMEM/F12 culture medium supplemented with 10% FBS was added. The plates were subsequently centrifuged at 1000× *g* for 10 min at 28 °C to form spheroids, and the cells were incubated for 7 days at the same conditions mentioned in Section 2.2. Half of the culture medium was carefully removed every 2 days, and a new medium was added to each well.

### 2.4. Treatment with Graphene Oxide and Dendrimers

The cells were plated in 35 mm dishes for the monolayer experiments at a density of 3 × 10^4^ cells/dish (Kasvi, São José Dos Pinhais, Brazil). After 48 h, graphene oxide (GOX), graphene oxide associated with dendrimer DAB-AM-16 (GOXD) and graphene oxide associated with dendrimer PAMAM (GOXP) were kept in the ultrasonic bath for 30 min. After that, the cells were exposed to the materials at concentrations of 24 μg/cm^2^ for 24 h, previously aliquoted for 24 h with the medium in the presence or absence of FBS. For the spheroid experiments, after 7 days of incubation, they were collected and moved to a 24-well flat bottom sterile cell culture plate (Kasvi, São José Dos Pinhais, Brazil). After 72 h, spheroids were treated with GOXP at concentrations of 24 μg/cm^2^ and images were taken in real time by the Lionheart microscope (Biotek, Winooski, VT, USA) for 48 h.

### 2.5. Immunofluorescence

This experiment was performed to evaluate structural changes in the cytoskeleton when cells were exposed to nanomaterials in the presence and absence of fetal bovine serum. For this, the cells were fixed with 3.7% formaldehyde in PBSA, and the plasma membrane was permeabilized with 0.5% Triton X-100 for 20 min. After that, they were incubated overnight with a mix of primary monoclonal anti-α and anti-β tubulin antibodies inside a moist chamber. After three washes with PBSA, the cells were incubated for 2 h with the fluorochrome-conjugated secondary anti-mouse antibody. Subsequently, F-actin were stained with Alexa 633-phalloidin for 60 min and the nuclei were stained with DAPI. Finally, the cytological preparations were mounted on the microscopic slide with Vectashield (Vector) and observed under the Lionheart (Biotek, Winooski, VT, USA) fluorescence microscope.

### 2.6. Mitosis Quantification

To evaluate the occurrence of mitosis in monolayer cells under the conditions mentioned in Section 2.4, the cells were fixed with 3.7% formaldehyde in PBSA after 24 h of the exposition of the GOX materials, the plasma membrane was permeabilized with 0.5% Triton X-100 for 20 min. After that, they were incubated overnight with a primary monoclonal anti-α tubulin antibody inside a moist chamber. On the following day, after three washes with PBSA, the cells were incubated for 2 h with the fluorochrome-conjugated secondary anti-mouse antibody. Subsequently, the nuclei were stained with Hoechst 33258 (Sigma, São Paulo, Brazil) for 30 min. Finally, the cytological preparations were mounted on the microscopic slide with Vectashield (Vector). Under the Lionheart (Biotek, Winooski, VT, USA) microscope, the nucleus area was determined and mitosis was quantified in at least 6 fields by preparations.

### 2.7. Real-Time Microscopy Cell Culture Monolayer and 3D Spheroids

The MCF-7 monolayer and MCF-7 3D spheroids were exposed to nanomaterials and the interaction of the particles with the cells was observed at the Lionheart (Biotek, Winooski, VT, USA) microscope coupled to a CO_2_ atmosphere, which provides the ideal conditions for the cells to remain alive. The images of spheroids were taken for 48 h at the same conditions.

### 2.8. Cellular Exposition to Fluorescent Graphene in the Presence of Internalization Inhibitors

The interaction between graphene oxide-based materials and the cells was analyzed in the presence of internalization pathway inhibitors. Previously, the cells were exposed to 2 h of treatments with the inhibitors, followed by one wash with PBSA and the addition of materials based on GOX (previously shaken for 12 h at 750 rpm with lophine NO_2_ to obtain fluorescence) with the same concentration of the previously used inhibitor for an additional 3 h. Subsequently, three washes with PBSA were performed and cells were fixed with 3.7% formaldehyde for 30 min. Preparations were mounted with Vectashield (Vector) and observed under a Lionheart (Biotek, Winooski, VT, USA) fluorescence microscope. Other cell treatments were also prepared without inhibitors with the same conditions described above with fluorescent graphene. They were kept in the refrigerator at 4 °C for the same length of time as they received the inhibitor treatments (2 h without graphenes, followed by 3 h with the fluorescent graphene). These treatments were named as total inhibition control (low temperature). We evaluated the effects of Chlorpromazine Hydrochloride (Sigma C8138) prepared in PBSA at 28.1 mM, at the final concentration of 28 µM in cells, for inhibition of clathrin-mediated endocytosis, as well as the effects of cytochalasin B (Sigma C6762) prepared in DMSO at 1 mg/mL, at a concentration of 2.5 µg/mL, to study the inhibition of macropinocytosis.

## 3. Results

### 3.1. The Presence of FBS in the Culture Medium Provided the Formation of Material Aggregates Avoiding the Formation of Large Particle Clusters

**Figure 1:**
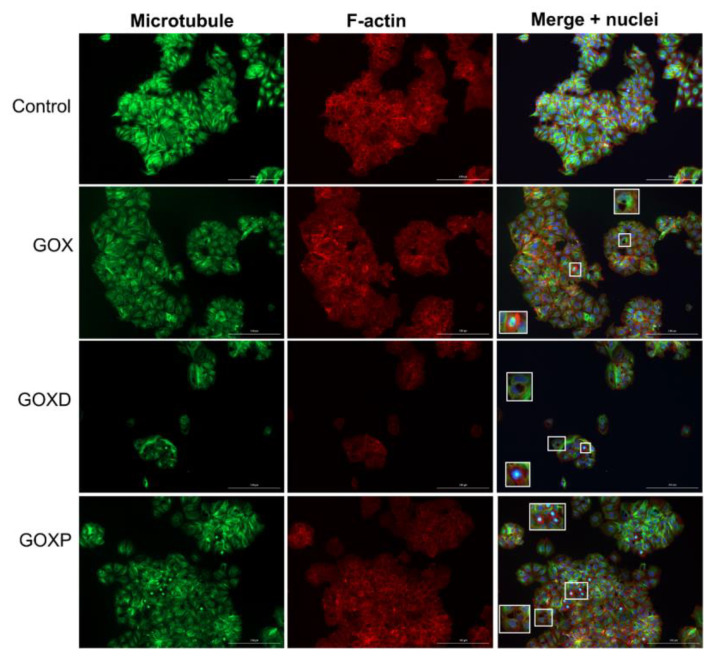
Immunofluorescence experiment of MCF-7 cells cultured in fetal bovine serum-containing medium treated with GOX, GOXD and GOXP at the concentration of 24 µg/cm^2^ for 24 h. The scale bar is 200 µm. The microtubules are evidenced by a mix of monoclonal antibodies against α and β tubulin and anti-mouse FITC-antibody (green). The microfilaments stained with Alexa 633-phalloidin (red). The nuclei were stained with DAPI (blue). Inserts are zooms (white rectangles) showing the occurrence of cell divisions and the presence of graphene clusters. Images acquired with the fluorescence microscope.

**Figure 2:**
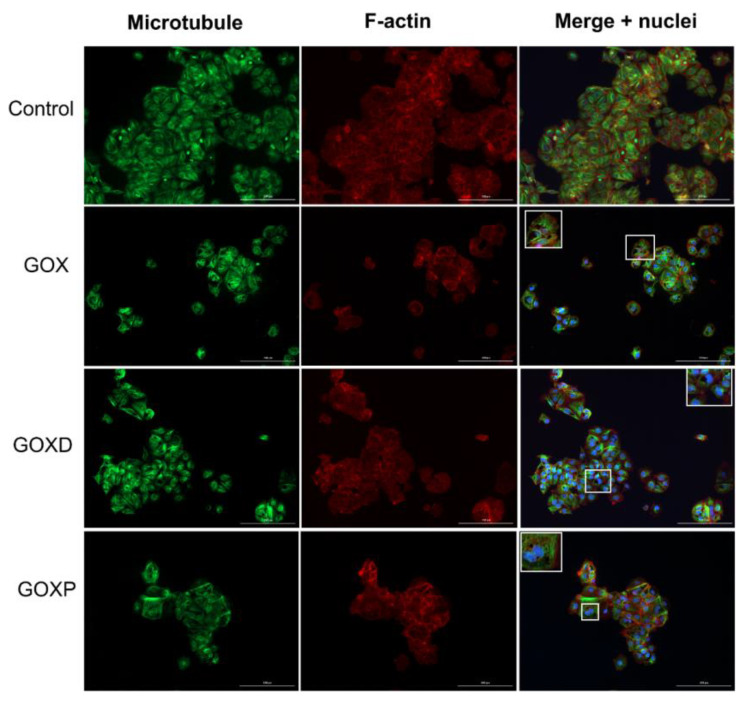
Immunofluorescence experiment of MCF-7 cells cultured in fetal bovine serum-free medium treated with GOX, GOXD and GOXP at the concentration of 24 µg/cm^2^ for 24 h. The scale bar is 200 µm. The microtubules are evidenced by a mix of monoclonal antibodies against α and β tubulin and anti-mouse FITC-antibody (green). The microfilaments stained with Alexa 633-phalloidin (red). The nuclei was stained with DAPI (blue). Inserts are zooms on white rectangles showing the presence of clusters. Images acquired with the Lionheart fluorescence microscope.

After observing that GOX, GOXD and GOXP were internalized by the MCF-7 cell line and caused morphological changes in the cells [25], it was sought to elucidate whether the presence of FBS in the culture medium would influence the internalization of materials and the frequency of mitosis. Then, immunofluorescence experiments were performed using antibodies to label the cytoskeleton. Firstly, MCF-7 cells were treated with GOX, GOXD and GOXP at a concentration of 24 µg/cm^2^ for 24 h. By analyzing the immunofluorescence preparations, it was observed that the graphene clusters were apparently more sparsely distributed throughout the cells when they were cultured in medium with fetal bovine serum (Figure 1 and Figure 2).

**Figure 3:**
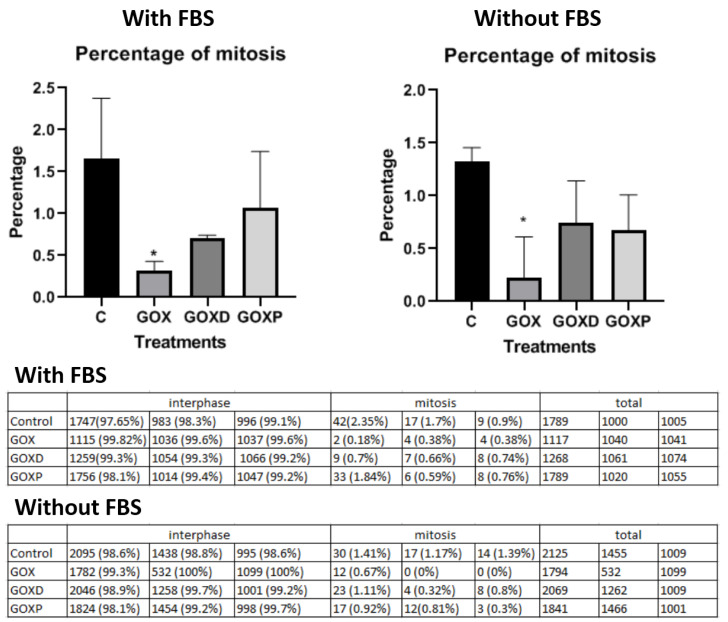
Quantification of mitosis in MCF-7 cells exposed to the GOX materials with FBS and without FBS. Statistical analysis was performed in GraphPad Prism and corresponds to ANOVA analysis. * *p* < 0.05.

In the mitosis quantification experiment, it was observed that in the presence and absence of serum, only the GOX material presented a significantly lower of mitosis compared to the control (Figure 3 and Supplementary Figure S1).

### 3.2. During Cell Division GOX, GOXD and GOXP Particles Were Redistributed to the Daughter Cells

**Figure 4:**
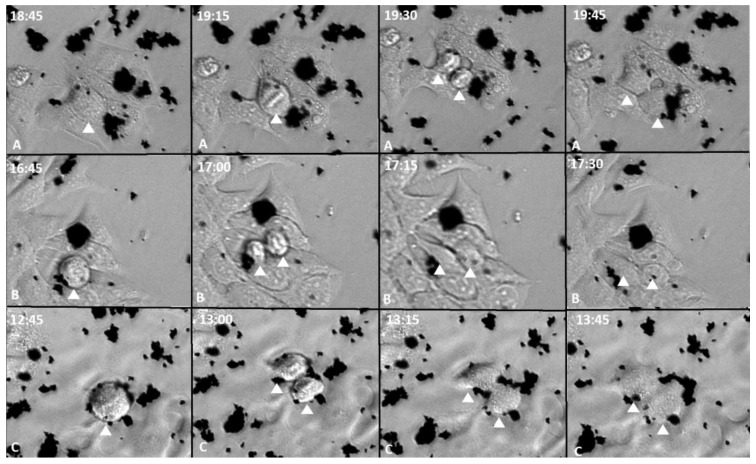
MCF-7 cells in 24 h of exposure to GOX, GOXD and GOXP at a concentration of 24 µg/cm^2^ in serum-free medium (A: GOX; B: GOXD; C: GOXP). Arrowhead: dividing cells. Images acquired with the Lionheart microscope. White numbers are the experimental time in hours.

**Figure 5:**
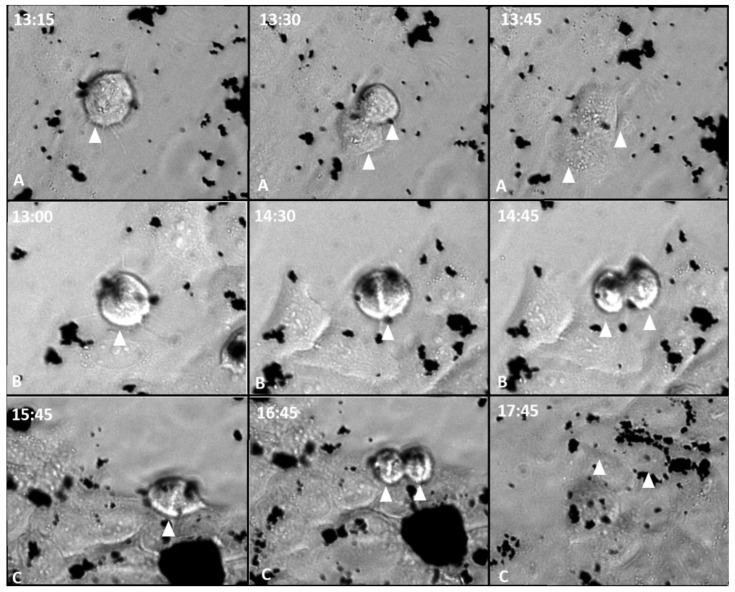
MCF-7 cells in 24 h of exposure to GOX, GOXD and GOXP at a concentration of 24 µg/cm^2^ in medium with 10% FBS (A: GOX; B: GOXD; C: GOXP). Arrowhead: mitotic cells. Images acquired with the Lionheart microscope. White numbers are the experimental time in hours.

It was analyzed if GOX, GOXD and GOXP were internalized by cells into large particles and, subsequently, redistributed, or if they were incorporated into small size and, within the cell, were brought together. With the real-time microscopy experiment, it was possible to observe that at each cell division, the cells with the internalized materials redistributed them to the daughter cells (Figure 4 and Figure 5) (all videos can be accessed in the Supplementary Materials), supporting the hypothesis that the cells incorporated the larger particles and were able to break them later. This mechanism characterizes these materials as an important biomedical tool for future applications.

### 3.3. GOXP Is also Redistributed in Cells Migrating from the Spheroid

**Figure 6:**
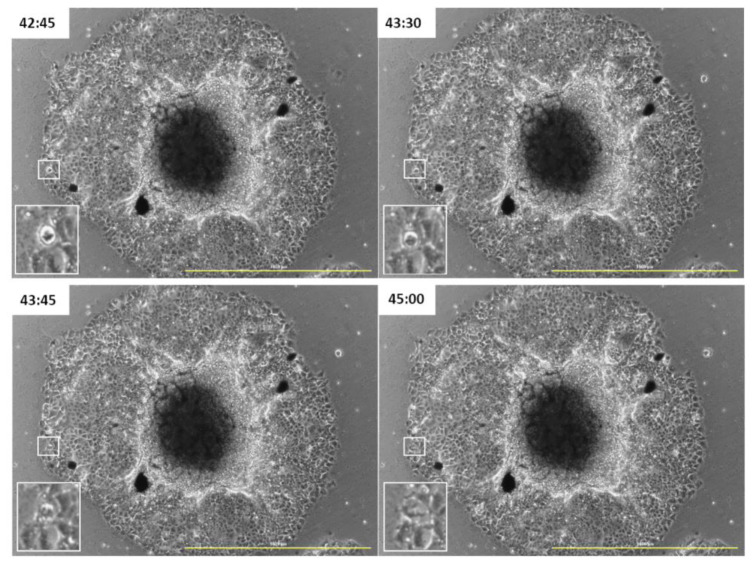
MCF-7 cells in 48 h of exposure to GOXP at a concentration of 24 µg/cm^2^ in medium with 10% FBS. Highlighted: mitotic cells. Images acquired with the Lionheart microscope. Scale bar: 1000 µm. The numbers are the experimental time in hours.

Interestingly, the cellular ability to redistribute GOXP to daughter cells in mitosis was also observed in migrating cells from spheroids (Figure 6). This result can be seen in the rectangles highlighted in the figure below. The 3D cell culture model mimics the in vivo environment more closely than just the monolayer cultures. The observation of this result, also in this model, emphasizes this material as an important biomedical tool for future applications.

### 3.4. GOXP Internalization Is Carried out by Clathrin-Mediated Endocytosis, While GOX and GOXD Are Internalized by Macropinocytosis

**Figure 7:**
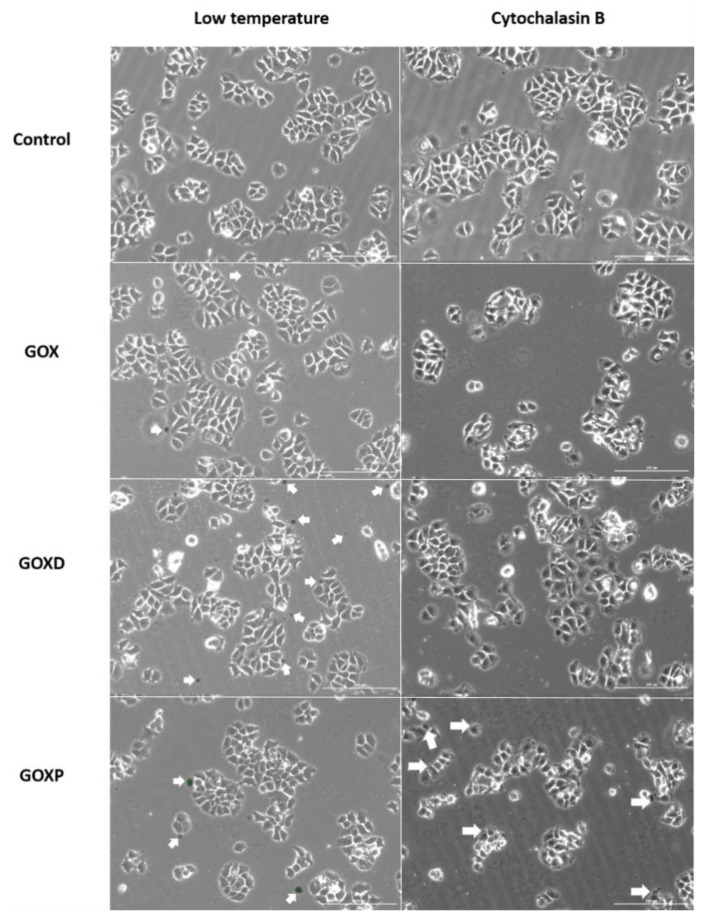
Images of MCF-7 cells obtained under the Lionheart fluorescence microscope. Low temperature corresponds to the cells that stayed in the refrigerator for 2 h. After that, cells received graphenes at the concentration of 24 µg/cm^2^ and lophines at a concentration of 1:1000 (previously shaken for 12 h at 750 rpm), for more than 3 h. In other dishes, cytochalasin B (2.5 µg/mL) was added for 2 h, cells were washed with fresh medium and graphene oxide (in the concentration 24 µg/cm^2^ and lophines 1:1000 of the concentration previously prepared for 12 h at 750 rpm) was added for 3 h in presence of the same inhibitor concentration. The arrows highlight the surface of the cells in interaction with the materials. Bar: 200 µm.

**Figure 8:**
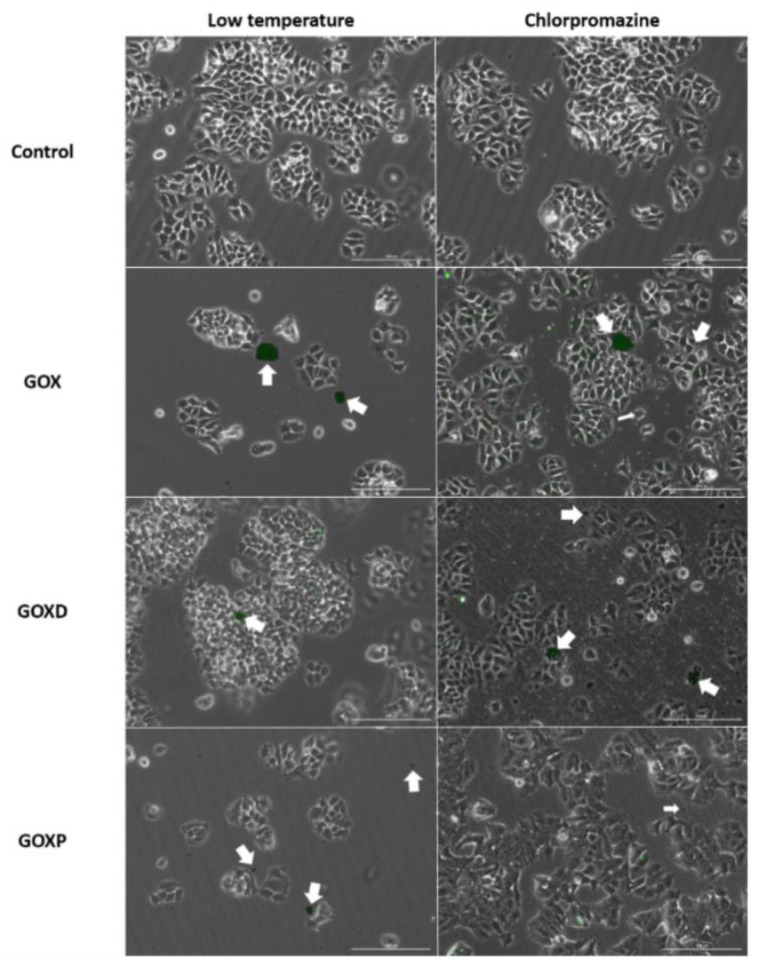
Images of MCF-7 cells obtained under the Lionheart fluorescence microscope. Low temperature corresponds to the cells that stayed in the refrigerator for 2 h. After that, cells received graphenes at the concentration of 24 µg/cm^2^ and lophines at a concentration of 1:1000 (previously shaken for 12 h at 750 rpm), for more than 3 h. In other dishes, Chlorpromazine Hydrochloride (28 µM) was added for 2 h, cells were washed with fresh medium and graphene oxide (in the concentration 24 µg/cm^2^ and lophines 1:1000 of the concentration previously prepared by shaked for 12 h at 750 rpm) was added for 3 h in presence of the same inhibitor concentration. The arrows highlight the surface of the cells in interaction with the materials. Bar: 200 µm.

The interaction of fluorescently labeled materials (obtained with lophine NO_2_) with MCF-7 cells was qualitatively analyzed by using different internalization inhibitors. In Figure 7 and Figure 8, it can be observed that a reduction of interaction between GOX and GOXD materials with cells was obtained with 2.5 µg/mL of cytochalasin B, which inhibits macropinocytosis. Less interaction between particles and cells was observed, as evidenced by the distribution distant from cells (Figure 7, highlighted by arrows). In GOXP, however, there was a decrease in interaction with cells with 28 µM of chlorpromazine, which inhibits clathrin-mediated endocytosis (Figure 8, highlighted by arrows).

## 4. Discussion

The results presented here showed that in the presence of a culture medium supplemented with fetal bovine serum, nanomaterials interacted more with the cells, which facilitated entry into the cells.

These results are similar to those reported by Bussy and Kostarelos (2017) [29] and Sun et al. (2016) [30]. They described that the effects of GOX on fibroblasts, A549, BEAS-2B, MDA-MB-231 and RBL cells were dependent on the composition of the culture medium. They also observed a profound plasma membrane (PM) ruffling and shedding in cells induced by GOX, and the appearance of intracellular vesicles. In this aspect of cellular response, Sima et al. (2020) [31] showed that fetal bovine serum was able to reduce particle cytotoxicity through the analysis of safe concentrations in tests by live/dead cell staining and MTS assays.

Malik et al. (2020) [32] showed that the interaction between proteins and different GOX samples was low and electrostatic, involving a rapid dynamic exchange of protein molecules on the surface of the particle. The authors saw that this interaction can be modulated through the degree of GOX oxidation, aiming for better stability in biomedical fields. It is also worth mentioning that when FBS proteins bind to the GOX surface and lead to the formation of the protein corona, it starts to govern cellular interactions instead of the GOX surface properties [33]. Thus, controlling the formation of the protein corona is still a challenging task, and allowing a prediction of the formation of the corona on nanoparticle surfaces and their internalization and, consequently, biodistribution are important tasks for the future of biomedical research [34].

In addition, in Figure 2, the particles showed a more concentrated distribution than in Figure 1, and this could happened precisely because of the interaction of these particles with the cytoskeleton resulting from this internalization process, considering that in the presence of the FBS the internalization of the particles could be more efficient. Another relevant observation is that without an organic surface coating, obtained by chemical modifications or due to adsorbed proteins, nanoparticles clump together [35]. In this context, the graft of hydrophilic polymers, which stabilize the nanoparticles, can reduce the adsorption of proteins on the surface. Wang et al. (2013) [36] showed that the protection provided by the protein coating is best observed when the particles are positively charged, since this is able to trigger cell death due to apoptosis.

Finally, an interaction of GOX, GOXD and GOXP with the cells was observed (Figure 4 and Figure 5). The mechanism of incorporation of the materials could depend on the cytoskeleton. This result characterizes macropinocytosis, in which the internalization of large amounts of extracellular material occurs by the activation of actin microfilaments to move the digestive vesicle. This explains the structural changes caused by the materials [37] and was confirmed qualitatively through the use of internalization inhibitors in GOX and GOXD (Figure 7). In GOXP, however, there was a qualitative decrease in interaction with cells with 28 µM of chlorpromazine, which inhibits clathrin-mediated endocytosis (Figure 8).

These results agree with the literature. Chatterjee et al. (2014) [38] observed by flow cytometry that in HepG2 cells, in the presence of endocytic pathway inhibitors, GOX was internalized by macropinocytosis and clathrin-mediated endocytosis. Furthermore, Huang et al. (2012) [39] described that human cervical carcinoma-derived cells in the presence of polyethylene glycol-modified GOX performed internalization primarily via clathrin-mediated endocytosis.

In addition, the results presented by real-time microscopy showed that materials based on graphene oxide have an important potential in the transport of drugs since, through their association with compounds, the cells would receive and distribute these materials at each cell division. This fact agrees with what was published by Bina et al. (2021) [40], who, through a computational study of molecular dynamics, described that the adsorption of compounds by materials based on graphene oxide occurs spontaneously and is more stable at neutral pH. Other studies have already found that modifying the surface of these materials with proteins significantly reduces cytotoxicity and can increase compound loading, confident of their efficiency for biomedical treatments. This could be due, in part, to the deposition of the protein corona, which is formed on the surface of nanoparticles and can optimize their efficiency [41].

Traditionally, these in vitro cell based assays are performed using two-dimensional (2D) monolayer cell culture, but the culturing of cells in a spheroid conformation increases the number cell–cell natural interactions, and if synthesizing their own extracellular matrices, by allowing cell-matrix interactions, these interactions mediate the behavioral and phenotypic characteristics of tissues and tumors, allowing cultures to mimic the in vivo situation in a more realistic and representative way [42].

When cultured in the monolayer, the cells receive the same amount of nutrients and growth factors from the medium in the culture dish. So, most of them are cycling, whereas 3D cells are often in different cell stages like a tumor cell in vivo. In 3D culture, it was observed that there were proliferating cells in the external region of the spheroid, surrounding the quiescent cells. The central cells of spheroids often remain quiescent as they receive less oxygen and growth factors from the environment. The oxygen, pH, and nutrient gradients are similar to those of the avascular regions of solid tumors in vivo [43,44].

Spheroids are suitable models to obtain additional information on nanoparticles and their use in cancer research; while they are not able to replace in vivo studies, they can bridge the gap between traditional 2D in vitro studies and in vivo models [45].

Our results are important to understand the behavior of mammalian carcinoma cells with nanomaterials, making the evaluation of therapeutic tools safer.

## 5. Conclusions

In conclusion, the present results in this study showed that the presence of the serum facilitated a more efficient internalization of the materials through the interaction of the particles with the cell membrane. Previous results performed for us showed that GOX, GOXD and GOXP are materials with interesting biomedical applications since they can be internalized by the cells. In addition, the particles could interact with the cell surface and with the cytoskeleton, favoring their internalization. Also, the behavior of the cells when internalizing the materials was reported, showing that they were able to redistribute them to the daughter cells, which characterizes them as an important therapeutic tool when associated with drugs. The LOT technique promoted the assembly of highly reproducible and individual 3D spheroids that can be used for future in vitro assays. This study proved to be a pioneer in the study of internalization by real-time microscopy.

## Data Availability

The datasets generated and/or analyzed during the current study are available from the corresponding author on reasonable request.

## Supplementary Materials

The following supporting information can be downloaded at: https://www.mdpi.com/article/10.3390/pharmaceutics15122655/s1, Figure S1: Immunofluorescence reaction of MCF-7 cells in culture fetal bovine serum-containing medium (above) and without FBS (below) exposed to GOX, GOXD and GOXP at the concentration of 24 µg/cm^2^ for 24 h. The scale bar is 100 µm. The microtubules are evidenced by a monoclonal antibody against α-tubulin and anti-mouse CY5-antibody (red). The nucleus of the cells were stained by Hoechst (blue). Arrows highlight mitosis. Images acquired with the Lionheart fluorescence microscope. Video S1: MCF-7 cells recorded for 24 h of treatment with GOX, at a concentration of 24 µg/cm² in medium without FBS (on the top) and medium with 10% FBS (below). Images acquired with the Lionheart microscope. Video S2: MCF-7 cells recorded for 24 h of treatment with GOXD, at a concentration of 24 µg/cm² in medium without FBS (on the top) and medium with 10% FBS (below). Images acquired with the Lionheart microscope. Video S3: MCF-7 cells recorded for 24 h of treatment with GOXP, at a concentration of 24 µg/cm² in medium without FBS (on the top) and medium with 10% FBS (below). Images acquired with the Lionheart microscope. Video S4: MCF-7 3D spheroids recorded for 48 h. On the top: cells without graphene oxide-based materials (control). Bellow: cells exposed to GOXP, at a concentration of 24 µg/cm² in culture medium with 10% FBS. Images acquired with the Lionheart microscope.

## References

[1] Novoselov K.S., Geim A.K., Morozov S.V., Jiang D., Zhang Y., Dubonos S.V., Grigorieva I.V., Firsov A.A. (2004). Electric field effect in atomically thin carbon films. Science.

[2] Geim A.K., Novoselov K.S. (2007). The rise of graphene. Nat. Mater..

[3] Balaji A., Yang S., Wang J., Zhang J. (2019). Graphene Oxide-Based Nanostructured DNA Sensor. Biosensors.

[4] Han X.-M., Zheng K.-W., Wang R.-L., Yue S.-F., Chen J., Zhao Z.-W., Song F., Su Y., Ma Q. (2020). Functionalization and optimization-strategy of graphene oxide-based nanomaterials for gene and drug delivery. Am. J. Transl. Res..

[5] Li J., Zeng H., Zeng Z., Zeng Y., Xie T. (2021). Promising Graphene-Based Nanomaterials and Their Biomedical Applications and Potential Risks: A Comprehensive Review. ACS Biomater. Sci. Eng..

[6] Dias A.P., da Silva Santos S., da Silva J.V., Parise-Filho R., Ferreira E.I., El Seoud O., Giarolla J. (2020). Dendrimers in the context of nanomedicine. Int. J. Pharm..

[7] Li H., Papadakis R. (2021). Click Chemistry Enabling Covalent and Non-Covalent Modifications of Graphene with (Poly)saccharides. Polymers.

[8] Sun X., Zhao P., Lin J., Chen K., Shen J. (2023). Recent advances in access to overcome cancer drug resistance by nanocarrier drug delivery system. Cancer Drug Resist..

[9] Rahman S., Kumar V., Kumar A., Abdullah T.S., Rather I.A., Jan A.T. (2021). Molecular Perspective of Nanoparticle Mediated Therapeutic Targeting in Breast Cancer: An Odyssey of Endoplasmic Reticulum Unfolded Protein Response (UPR^ER^) and Beyond. Biomedicines.

[10] Pourjavadi A., Asgari S., Hosseini S.H., Akhlaghi M. (2018). Codelivery of Hydrophobic and Hydrophilic Drugs by Graphene-Decorated Magnetic Dendrimers. Langmuir.

[11] Lyu Z., Ding L., Huang A.-T., Kao C.-L., Peng L. (2019). Poly(amidoamine) dendrimers: Covalent and supramolecular synthesis. Mater. Today Chem..

[12] Lyu Z., Ding L., Tintaru A., Peng L. (2020). Self-assembling supramolecular dendrimers for biomedical applications: Lessons learned from poly(amidoamine) dendrimers. Acc. Chem. Res..

[13] Marichal L., Giraudon--Colas G., Cousin F., Thill A., Labarre J., Boulard Y., Aude J.-C., Pin S., Renault J.P. (2019). Protein–nanoparticle interactions: What are the protein–corona thickness and organization?. Langmuir.

[14] Vianello F., Cecconello A., Magro M. (2021). Toward the Specificity of Bare Nanomaterial Surfaces for Protein Corona Formation. Int. J. Mol. Sci..

[15] Simon J., Müller L.K., Kokkinopoulou M., Lieberwirth I., Morsbach S., Landfester K., Mailänder V. (2018). Exploiting the biomolecular corona: Pre-coating of nanoparticles enables controlled cellular interactions. Nanoscale.

[16] Monteiro A.R., Neves M.G.P.M.S., Trindade T. (2020). Functionalization of Graphene Oxide with Porphyrins: Synthetic Routes and Biological Applications. ChemPlusChem.

[17] Vacchi I.A., Guo S., Raya J., Bianco A., Ménard-Moyon C. (2020). Strategies for the Controlled Covalent Double Functionalization of Graphene Oxide. Chem. A Eur. J..

[18] Jayaram D.T., Pustulka S.M., Mannino R.G., Lam W.A., Payne C.K. (2018). Protein corona in response to flow: Effect on protein concentration and structure. Biophys. J..

[19] Liu N., Tang M., Ding J. (2020). The interaction between nanoparticles-protein corona complex and cells and its toxic effect on cells. Chemosphere.

[20] Pinals R.L., Yang D., Rosenberg D.J., Chaudhary T., Crothers A.R., Iavarone A.T., Hammel M., Landry M.P. (2020). Quantitative Protein Corona Composition and Dynamics on Carbon Nanotubes in Biological Environments. Angew. Chem. Int. Ed..

[21] Behzadi S., Serpooshan V., Tao W., Hamaly M.A., Alkawareek M.Y., Dreaden E.C., Brown D., Alkilany A.M., Farokhzad O.C., Mahmoudi M. (2017). Cellular uptake of nanoparticles: Journey inside the cell. Chem. Soc. Rev..

[22] Åberg C., Piattelli V., Montizaan D., Salvati A. (2021). Sources of variability in nanoparticle uptake by cells. Nanoscale.

[23] Rennick J.J., Johnston A.P.R., Parton R.G. (2021). Key principles and methods for studying the endocytosis of biological and nanoparticle therapeutics. Nat. Nanotechnol..

[24] Varma S., Dey S., Palanisamy D. (2022). Cellular Uptake Pathways of Nanoparticles: Process of Endocytosis and Factors Affecting their Fate. Curr. Pharm. Biotechnol..

[25] Ribeiro B.F.M., Souza M.M., Fernandes D.S., Carmo D.R.D., Machado-Santelli G.M. (2020). Graphene oxide-based nanomaterial interaction with human breast cancer cells. J. Biomed. Mater. Res. Part A.

[26] Fernandes D.S., Bonfim K.S., Carmo D.R.D. (2018). Silver Hexacyanoferrate (III) on a Hybrid Graphene Oxide/PAMAM Dendrimer Surface and Application as an Electrocatalyst in the Detection of Isoniazid. Electroanalysis.

[27] Carmo D.R.D., Fernandes D.S. (2017). Hybrid graphene oxide/DAB-Am-16 dendrimer: Preparation, characterization chemical reactivity and their electrocatalytic detection of l-Dopamine. Solid State Sci..

[28] Wu B., Xiao L., Zhang M., Yang C., Li Q., Li G., He Q., Liu J. (2021). Facile synthesis of dendritic-like CeO_2_/rGO composite and application for detection of uric acid and tryptophan simultaneously. J. Solid State Chem..

[29] Bussy C., Kostarelos K. (2017). Culture media critically influence graphene oxide effects on plasma membranes. Chem.

[30] Sun C., Wakefield D.L., Han Y., Muller D.A., Holowka D.A., Baird B.A., Dichtel W.R. (2016). Graphene oxide nanosheets stimulate ruffling and shedding of mammalian cell plasma membranes. Chem.

[31] Sima L.E., Chiritoiu G., Negut I., Grumezescu V., Orobeti S., Munteanu C.V.A., Sima F., Axente E. (2020). Functionalized Graphene Oxide Thin Films for Anti-tumor Drug Delivery to Melanoma Cells. Front. Chem..

[32] Malik S.A., Mohanta Z., Srivastava C., Atreya H.S. (2020). Modulation of protein–graphene oxide interactions with varying degrees of oxidation. Nanoscale Adv..

[33] Franqui L.S., De Farias M.A., Portugal R.V., Costa C.A., Domingues R.R., Filho A.G.S., Coluci V.R., Leme A.F., Martinez D.S.T. (2019). Interaction of graphene oxide with cell culture medium: Evaluation the fetal bovine serum protein corona formation towards in vitro nanotoxicity assessments and nanobiointeractions. Mater. Sci. Eng. C Mater. Biol. Appl..

[34] Partikel K., Partikel K., Korte R., Korte R., Mulac D., Mulac D., Humpf H.-U., Humpf H.-U., Langer K., Langer K. (2019). Serum type and concentration both affect the protein-corona composition of PLGA nanoparticles. Beilstein J. Nanotechnol..

[35] Rivera-Gil P., De Aberasturi D.J., Wulf V., Pelaz B., Del Pino P., Zhao Y., De La Fuente J.M., De Larramendi I.R., Rojo T., Liang X.-J. (2013). The Challenge To Relate the Physicochemical Properties of Colloidal Nanoparticles to Their Cytotoxicity. Acc. Chem. Res..

[36] Wang F., Yu L., Monopoli M.P., Sandin P., Mahon E., Salvati A., Dawson K.A. (2013). The biomolecular corona is retained during nanoparticle uptake and protects the cells from the damage induced by cationic nanoparticles until degraded in the lysosomes. Nanomed. Nanotechnol. Biol. Med..

[37] Dąbrowski B., Żuchowska A., Brzózka Z. (2023). Graphene oxide internalization into mammalian cells—A review. Colloids Surf. B Biointerfaces.

[38] Chatterjee N., Eom H.-J., Choi J. (2014). A systems toxicology approach to the surface functionality control of graphene–cell interactions. Biomaterials.

[39] Huang J., Zong C., Shen H., Liu M., Chen B., Ren B., Zhang Z. (2012). Mechanism of cellular uptake of graphene oxide studied by surface-enhanced raman spectroscopy. Small.

[40] Bina A., Raissi H., Hashemzadeh H., Farzad F. (2021). Conjugation of a smart polymer to doxorubicin through a pH-responsive bond for targeted drug delivery and improving drug loading on graphene oxide. RSC Adv..

[41] Sebak A.A., Gomaa I.E.O., ElMeshad A.N., Farag M.H., Breitinger U., Breitinger H.-G., AbdelKader M.H. (2020). Distinct Proteins in Protein Corona of Nanoparticles Represent a Promising Venue for Endogenous Targeting—Part I: In vitro Release and Intracellular Uptake Perspective. Int. J. Nanomed..

[42] Jensen C., Teng Y. (2020). Is it time to start transitioning from 2D to 3D cell culture?. Front. Mol. Biosci..

[43] Amaral J.B.D., Rezende-Teixeira P., Freitas V.M., Machado-Santelli G.M. (2011). MCF-7 cells as a three-dimensional model for the study of human breast cancer. Tissue Eng. Part C Methods.

[44] Millard M., Yakavets I., Zorin V., Kulmukhamedova A., Marchal S., Bezdetnaya L. (2017). Drug delivery to solid tumors: The predictive value of the multicellular tumor spheroid model for nanomedicine screening. Int. J. Nanomed..

[45] Lu H., Stenzel M.H. (2018). Multicellular tumor spheroids (MCTS) as a 3D in vitro evaluation tool of nanoparticles. Small.

