# Supplementary figures and images for: Interaction of Graphene Oxide Particles and Dendrimers with Human Breast Cancer Cells by Real-Time Microscopy
Source: Pharmaceutics. 2023 Nov 22;15(12):2655. doi: 10.3390/pharmaceutics15122655 (PMC10747174; doi:10.3390/pharmaceutics15122655)

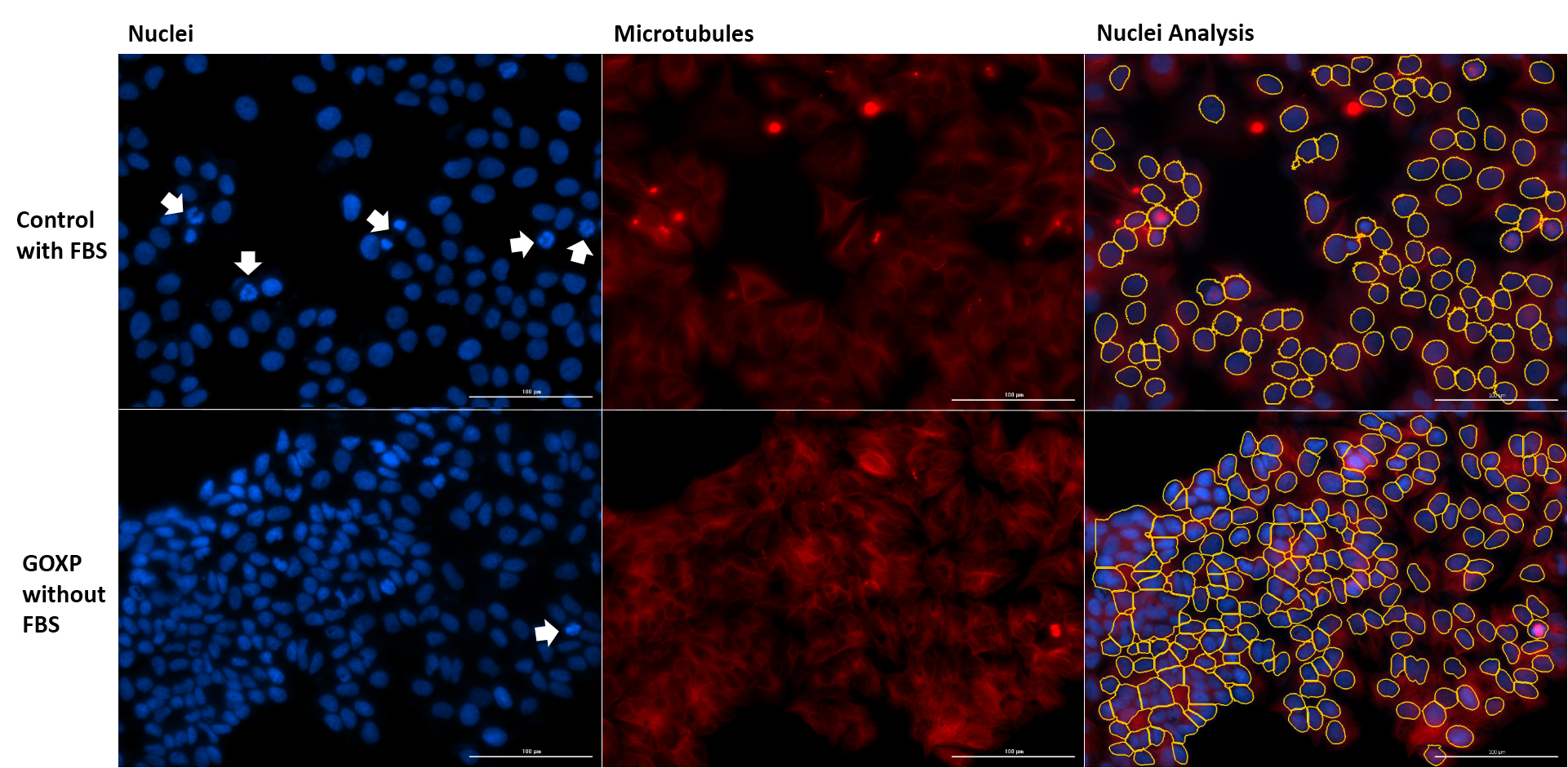

Supplement: Supplementary file 1 [file pharmaceutics-15-02655-s001.zip › pharmaceutics-2666119-supplementary/Suppl/Figure S1.tif]
